# Influenza infection of the mammary gland

**DOI:** 10.1128/jvi.01940-24

**Published:** 2025-08-12

**Authors:** Alyson A. Kelvin, Pari H. Baker, Sourish Ghosh, Stacey Schultz-Cherry, Stephanie N. Langel

**Affiliations:** 1 Faculty of Veterinary Medicine, University of Calgary 70404, Calgary, Alberta, Canada; 2 Department of Pathology, Center for Global Health and Diseases, Case Western Reserve University School of Medicine 12304, Cleveland, Ohio, USA; 3 Infectious Diseases & Immunology Division, CSIR - Indian Institute of Chemical Biology 30156, Kolkata, West Bengal, India; 4 Department of Host-Microbe Interactions, St Jude Children’s Research Hospital 5417, Memphis, Tennessee, USA; Indiana University Bloomington, Bloomington, Indiana, USA

**Keywords:** highly pathogenic avian influenza, H5N1 clade 2.3.4.4b, mammary gland, milk, dairy cattle, influenza, One Health

## Abstract

The mammary gland is an essential organ for milk production, providing essential immune and nutritional support to offspring and supplying dairy products for human consumption. In both humans and animals, the lactating mammary gland is susceptible to bacterial and viral infections, which can lead to mastitis and, in some cases, vertical transmission to offspring, with potential adverse effects on infant health. However, until recently, the role of respiratory viruses in mammary gland infection has been relatively understudied, particularly their ability to infect mammary epithelial cells and transmit through lactation. The recent emergence of highly pathogenic avian influenza H5N1 clade 2.3.4.4b in dairy cattle has demonstrated the virus’s capacity to replicate in the mammary gland, cause mastitis, and produce high viral loads in milk. This raises significant concerns about the potential for zoonotic transmission to humans and other animals in contact with infected dairy cows and unpasteurized milk. In this mini-review, we highlight key studies that demonstrate the replication of influenza and other viruses in the mammary gland, summarize recent findings from experimental and natural H5N1 clade 2.3.4.4b infections in dairy cows and small animal models, and discuss the broader One Health implications of the current H5N1 outbreak. We emphasize the urgent need for an interdisciplinary collaboration across sectors to mitigate the risks posed by influenza viruses with pandemic potential.

## INTRODUCTION

The mammary gland is a specialized organ for producing milk, which nourishes offspring and provides critical immunological factors for early development (1, 2). During pregnancy, the mammary gland undergoes significant physiological changes including increased blood flow and extensive elongation and branching of the mammary epithelium (3). These changes prepare the gland for lactation, during which milk components are provided as colostrum during the first 48 hours postpartum and as mature milk after (4–6). The dynamic exchange between the mother and infant during suckling provides an opportunity for pathogens to transfer either to the infant or back to the mammary gland via retrograde flow (7). In agricultural species such as dairy cows, which supply large quantities of milk for the food industry rather than a suckling neonate, mechanical milking and environmental exposure contribute to an increased risk of infection (8). Controlling pathogen exposure in lactating mothers or milk-supplying animals is critical for protecting maternal and neonatal health and ensuring a safe food supply.

Viruses are well known to infect cells within the lactating mammary gland and can be transmitted to infants. Notable examples in humans include human immunodeficiency virus (HIV) and cytomegalovirus (CMV). Additionally, members of the flavivirus family have been identified in human breast milk, including Zika virus (9), dengue virus (10, 11), and yellow fever virus (12–14). In animal studies, milk-associated virus transmission has been observed for Powassan virus in an experimental infection of a goat (15), West Nile virus in rodent models (16, 17), tick-borne encephalitis virus in cows and goats (18, 19), and Langat virus in a mouse model (20). Ghosh and colleagues demonstrated in a mouse model that enteric viruses, rotavirus and norovirus, can replicate in the mammary gland and be transmitted to suckling neonates (21). Additionally, Middle East Respiratory Coronavirus (MERS-CoV) has been detected in camel milk (22, 23), and Foot-and-Mouth Disease virus (FMDV) can replicate in the bovine mammary gland (24, 25).

Influenza virus, typically associated with respiratory infections, has not historically been associated with replication in the mammary gland or transmission through breastfeeding. However, in 2015, we demonstrated that H1N1 influenza virus can infect the lactating mammary glands of ferrets and transmit to offspring through breastfeeding (26), corroborating earlier reports suggesting that H1N1 can also infect the mammary glands of lactating dairy cattle (27). More recently, in March 2024, highly pathogenic avian influenza H5N1 clade 2.3.4.4b emerged in lactating dairy cattle, displaying a marked tropism for the mammary gland. Infected cows developed mastitis and harbored high viral titers in their milk (28–30). The mastitis was characterized by yellow, clotted milk, negatively impacting both milk quality and yield. The rapid spread of H5N1 clade 2.3.4.4b across dairy herds, now confirmed in dairy cattle across 17 states (as of July 2025) (31), raises concerns about the potential for zoonotic transmission to humans and animals with exposure to infected cattle and unpasteurized milk (32, 33).

Understanding viral pathogenesis in the mammary gland and the potential for transmission through expressed milk is essential not only for protecting mammary health but also for preventing broader outbreaks. Significant knowledge gaps remain regarding the mechanisms of viral replication in the mammary gland and the pathways by which viruses are transmitted to neonates, other animals, or the environment. In this review, we will discuss the implications of viral infection on lactation, mammary gland immune responses, and cross-species transmission risks, with a particular focus on influenza viruses. We also revisit historical and underrecognized evidence of influenza virus infection in the mammary gland and contextualize these findings considering the recent H5N1 outbreak in lactating dairy cattle.

## MAMMARY GLAND BIOLOGY AND IMPLICATIONS FOR VIRAL INFECTIONS

**Fig 1:**
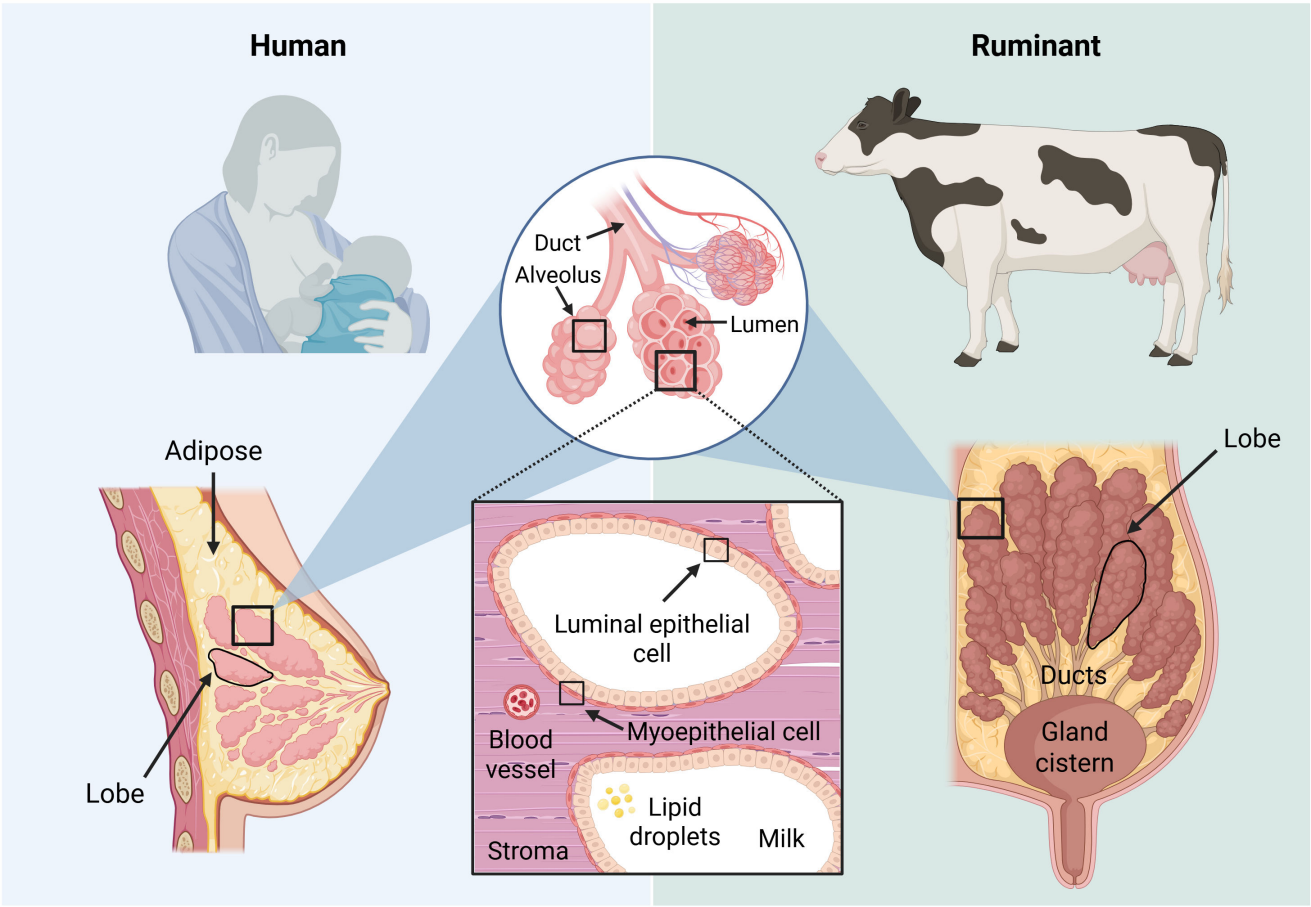
Comparative anatomy of human (left) and bovine (right) mammary gland structures.

Structurally, the mammary gland in both humans and cattle consists of two main tissue types: supportive stromal tissue, which includes a matrix of adipocytes, fibroblasts, lymphatics, blood vessels, and immune cells, and parenchymal tissue, such as luminal epithelial cells and myoepithelial cells (Fig. 1) (3). Luminal epithelial cells line the alveoli and ducts, producing and secreting milk in response to prolactin, a hormone produced by lactotroph cells in the anterior pituitary gland (2, 34). Myoepithelial cells surround these structures, contracting to facilitate milk ejection in response to oxytocin (35). Luminal epithelial cells are highly metabolically active and are responsible for synthesizing, storing, and secreting milk components such as proteins, lipids, and lactose during lactation (36). Their heightened metabolic activity and robust secretory capacity may render milk-producing epithelial cells especially vulnerable to viral replication as viruses readily exploit the abundant cellular machinery and energy resources inside cells (37). In humans, the mammary system consists of two mammary glands, while in dairy cows, the udder contains four independent mammary glands, enabling significantly higher milk production (Fig. 1). However, this higher milk output can also create conditions that favor pathogen growth, increasing the risk of disease transmission among dairy cattle.

The lactating mammary gland is not only a potential site for active viral replication but can also serve as a reservoir for viruses replicating in distant tissues. Due to its rich vascularization and extensive network of epithelial and immune cells, the mammary gland is susceptible to viral entry from other parts of the body. In humans, viruses such as HIV and CMV can disseminate through the bloodstream or lymphatic system (38, 39), establishing a reservoir within the gland that could lead to viral shedding into breast milk, posing a risk of transmission to offspring or others. Overall, understanding the barrier functions of the mammary gland is crucial for elucidating viral transmission mechanisms to suckling neonates or preventing environmental contamination in agricultural settings.

## INFLUENZA VIRUS AND SIALIC ACIDS

Influenza viruses (Orthomyxoviridae) are enveloped viruses with a negative-sense single-stranded segmented genome (40). The family is composed of four genera, types A, B, C, and D, where strains of types A and B circulate annually in humans (40–43). IAVs are of particular concern due to their association with more severe disease, their high genetic diversity within wild migratory bird reservoirs, and their substantial potential for spill over into other animal species, including humans. IAVs are defined by their immunogenic proteins hemagglutinin (HA) and neuraminidase (NA) on the surface of each virion. There are 19 different HAs and 11 NAs associated in various combinations to designate influenza subtypes such as H1N1, H3N2, or H5N1 (40, 44, 45). HA subtypes 1 through 16 mediate viral entry by binding to sialic acid (SA) receptors on host cells, specifically those presenting α2,6-linked sialic acid (α2,6 SA) or α2,3-linked (α2,3 SA) sialic acid. Sialic acids can be modified at various positions, such as by 5-N-acetylation, 5-N-glycolylation, or 9-O-acetylation, depending on the host species, and these modifications can influence viral binding and host specificity. Influenza viruses bearing HA subtypes H1–H16 occur as low pathogenic avian influenza (LPAI), with H5 and H7 subtypes also occurring as highly pathogenic avian influenza (HPAI) strains (46). LPAI viruses typically target epithelial cells of the respiratory tract in humans—similar to seasonal influenza viruses—and in other mammals, or the gastrointestinal tract in their avian reservoir hosts. Importantly, unlike seasonal LPAI strains, HPAI viruses such as H5N1 are capable of systemic spread in the body, efficiently infecting extra-respiratory tissues including the intestines and brain. This broader tissue tropism contributes to the more severe disease manifestations and multi-organ involvement observed in HPAI infections (47, 48).

The localization of influenza virus infection is largely determined by the distribution of the viral entry receptors α2,6 SA and α2,3 SA on target cells, as well as by specific sialic acid modifications. In humans, seasonal influenza viruses preferentially bind α2,6 SA, which is predominantly expressed in the upper respiratory tract, while avian influenza viruses such as H5N1 preferentially bind α2,3 SA, which is more abundantly expressed in the lower respiratory tract (49). In addition to the receptor distribution, the spread of infection is influenced by HA cleavage, a prerequisite for viral fusion and entry. The multibasic cleavage site (MBCS), enriched with arginine and lysine residues and present in HPAI viruses, is a known virulence factor associated with systemic infection in both poultry and mammals (50–52). The presence of multiple basic amino acids found around the cleavage site in HPAI viruses allows recognition by subtilisin-like proteases such as furin, which are ubiquitously expressed in cells of many tissues, allowing the expanded HPAI H5N1 tropism (53). In humans, mammals, and birds, HPAI viruses—H5N1 in particular—have been shown to infect extrapulmonary tissues, including the eyes, brain, intestines, placenta, and spleen and, more recently, the mammary gland. This expanded tropism is supported by the presence of SAs in these tissues and the capacity of MBCS in HPAI viruses to facilitate HA activation outside the respiratory tract (54, 55).

The recent emergence of H5N1 HPAI in lactating dairy cattle, marked by high levels of viral secretion in milk, has spurred further investigation into the influenza virus’s tropism for the mammary gland. Lectin staining of mammary gland tissues from dairy cattle with *Maackia amurensis* lectin II (MAL-II) revealed abundant expression of the H5N1 receptor, α2,3 SA, particularly on epithelial cells lining the secretory alveoli, whereas receptor expression in the trachea, bronchi, and pulmonary alveoli was low or undetectable (56–58). To confirm HA binding, Ríos Carrasco and colleagues demonstrated that HA from A/duck/France/161108 h/2016—a clade 2.3.4.4b virus with minimal amino acid differences from recent North American mammalian-derived strains—exhibited intense binding in mammary gland tissues. In contrast, HA proteins from classical H5 viruses with more extensive amino acid divergence, such as A/Vietnam/1203/2004 and A/Indonesia/05/2005, showed variable binding, while a clade 2.3.4.4 a virus displayed no detectable binding (57). These findings suggest that subtle structural variations in α2,3-linked sialic acids on bovine mammary epithelial cells, together with milk proteases such as plasmin (59) in bovine milk (60), may facilitate binding and replication of newer HPAI H5N1 strains.

While previous studies have detected α2,6 SA in the lactating bovine mammary gland using the lectin Sambucus nigra agglutinin (SNA) (56, 58), Ríos Carrasco et al. did not observe binding of HA from human-adapted H1N1 virus A/Puerto Rico/8/1934 (PR8) (57). This may suggest that α2,6 SAs in cattle do not serve as functional receptors for human-adapted IAVs potentially due to having a modification such as 5-N-glycolyl, which does not support binding of a humanized virus. However, *in vivo* evidence indicates that human-adapted H1N1 strains, including PR8 and A/California/07/2009, replicated in the lactating mammary glands of both dairy cows (27) and ferrets (26), as detailed below. These findings underscore the need for *in vivo* experimental infection studies of the mammary gland to more precisely define its susceptibility to IAVs.

## MAMMARY GLAND SUSCEPTIBILITY TO INFLUENZA VIRUS INFECTION

After the discovery of the influenza virus in 1933, researchers sought a method to expand the virus for experimental studies and vaccine development. A group in Quebec, Canada, suggested that the bovine mammary gland might support virus propagation due to dairy cows being selectively bred for high milk production and having enzymes needed for viral replication (27). To test this hypothesis, Mitchell and colleagues examined whether an ancestral human H1N1 influenza virus (now referred to as A/Puerto Rico/8/1934 (PR8) virus strain) could infect and replicate in cow mammary glands. To administer the virus, all milk was removed from the quarters, and 2.0 cc of egg-grown virus was introduced directly into the mammary gland’s lactiferous sinus (cistern) in the right front quarter using a teat needle through the teat canal. Milk samples from each quarter were collected daily to determine if virus was being shed as an indication of productive infection. As a control, adjacent quarters in the same cow that were not virus-inoculated were also milked and subjected to the same virological assays. Upon collection, milk was centrifuged and incubated at 4°C to remove the cream (fat), and the semi-transparent phase was examined for virus by inoculation into the allantoic fluid of chicken eggs. Initial studies demonstrated that PR8 could replicate within the bovine mammary gland and be shed into expressed milk, as confirmed by Egg Infectious Dose 50 (EID_50_). The viral titer of PR8 fluctuated, showing an increase on day 2 that was present at variable levels for another 9 days. The virus was cleared from the inoculated gland by day 14, following peak titers of 10^6^ EID_50_ in the milk. Uninoculated quarters were not found to be positive for virus, which agrees with previous literature that the bovine mammary glands are separated by connective tissue (61), preventing the direct spread of infection between quarters. The virus was not detected in blood samples, indicating the infection remained localized and did not spread to other quarters or tissues.

Blood and milk samples collected from infected cattle were investigated for the presence of virus-neutralizing antibodies. Virus-neutralizing antibodies were detected 14 days post-inoculation using the hemagglutinin inhibition assay (HAI), and their presence was further confirmed at 18 months post-infection using the chick embryo test (62–64). No samples were positive for both virus and antibodies on the same day, which the authors suggest may indicate that the elicitation of neutralizing antibodies plays a role in viral clearance (63). Interestingly, neutralizing antibodies in the uninoculated quarter were not present until 6 days after the virus was cleared from the inoculated gland. Secondary inoculations of the virus into the previously infected mammary gland failed to establish a successful infection, suggesting the development of local protective immunity within the gland.

The 1950 cattle studies above suggested the susceptibility of the mammary gland to influenza virus infection. However, prior to 2025, only the H1N1 virus had been directly linked to mammary gland infection in lactating dairy cattle, leaving the susceptibility of the mammary gland to other influenza A viral subtypes largely unknown. Notably, retrospective analyses published in the early 2000s reported reduced milk production in dairy cattle that were seropositive for H1N1 and H3N2 (65, 66). While indirect, these findings highlight the need to investigate bovine susceptibility to diverse influenza A virus subtypes—both to protect the dairy industry and to reduce the risk of viral reassortment and cross-species transmission.

### Influenza virus infection and transmission in lactating ferrets

In 2015, our group investigated the immune mechanisms influencing the severe outcomes of 2009 H1N1 influenza virus infection in neonates, utilizing the ferret model (26). To investigate age-related influenza pathogenesis in ferrets, we established a breeding colony that provided access to influenza-free ferrets at various ages and stages of development. We used 4-week-old ferret kits from our colony for these studies, which required co-housing the kits with their entire litter, including their lactating mothers. These mothers provided milk as the sole source of nourishment for the ferret kits.

What began as a pathogenesis study in ferret kits quickly evolved into an investigation of influenza virus transmission, with an unexpected focus on the mammary gland. Ferret kits were inoculated intranasally with 10^5^ EID_50_ of A/California/07/2009 (Cal/07), resulting in high nasal wash viral titers that peaked on day 2 post-inoculation. This was accompanied by significant clinical disease, with all ferret kits reaching humane endpoints by day 8. Mothers shed virus from their nares and had an increase in temperature by days 3 and 4 post ferret kit inoculation. These results indicated that the virus was transmitted from the ferret kit to the mother’s respiratory tract. While respiratory transmission is crucial, we also recognized that the mammary gland serves as a frequent contact point between the ferret mother and her kits. This raised the question of whether the virus could be transmitted to the mammary glands during nursing, and whether the gland or expressed milk might contain infectious virus or viral antigens. By day 4 post-kit inoculation, analysis of milk and mammary gland tissues revealed infectious virus in at least two mammary glands (8 out of 12 total glands) and one teat per lactating ferret from the infected litters. Additionally, expressed milk tested positive for infectious influenza virus by day 6, suggesting viral shedding into the expressed milk. Immunohistochemistry confirmed the presence of viral antigens in the mammary gland, showing peak levels of influenza viral nucleoprotein (NP) by day 7. The presence of viral protein coincided with significant destruction of milk-producing epithelial cells in the mammary gland. Furthermore, infected mammary glands showed cessation of milk production, signatures of glandular involution, reduced STAT5 protein levels, and nuclear recruitment of STAT3 in mammary epithelial cells (26). Additionally, we observed transcriptional changes resembling those associated with breast cancer progression, suggesting that the long-term health impacts of acute mammary gland infections warrant further investigation (26).

To confirm the susceptibility of the mammary gland to influenza virus infection and the gland’s ability to support viral replication, the lactating mammary glands of mother ferrets were directly inoculated with 10^5^ EID_50_ of Cal/07 into the lactiferous ducts (26). Inoculation resulted in infectious viral shedding in the milk and positive viral detection in the mammary glands on days 2 and 4 post-inoculation. Additionally, mothers with virus-inoculated glands developed clinical signs of disease, including elevated temperatures on day 2 post-inoculation and weight loss, indicating that illness resulted from direct infection of the mammary gland. Interestingly, later in the course of infection, ferret kits also developed clinical disease, with weight loss progressing to humane endpoints by day 7 following mammary gland inoculation. Ferret kits also exhibited respiratory tract infection, with infectious virus detected in their nasal washes on day 4 post-mammary gland inoculation, while the nasal washes of the mothers did not test positive until day 7. These results suggest that the mother’s respiratory tract was not infected by the mammary gland inoculation but instead by respiratory contact or aerosol transmission from the infected ferret kits. Furthermore, the earlier onset of respiratory tract infection in ferret kits compared to their mothers suggests that ingestion of virus-positive milk may have led to respiratory infection, a route not previously described for influenza virus transmission.

### Dairy cattle outbreak and role of mammary gland infection

In December 2021, H5N1 clade 2.3.4.4b viruses were first detected in North America in poultry and free-living gulls in St. Johns Newfoundland, Canada (67). Since then, viruses of this clade from this introduction and others have spread across all wild bird migratory flyways in the continent. Continental spread has led to spillover infections in an unprecedented number of mammalian species, including to dairy cattle (68). Surprisingly, spillover to dairy cattle led to onward transmission, causing an expansive outbreak on dairy farms in the United States (U.S.) and facilitating further farm-to-farm spread. As of this writing (July 2025), there are over 1,078 herds affected across 17 U.S. states (69, 70). Multiple introductions into dairy cattle are now hypothesized, as both B3.13 and D1.1 genotypes have been identified in positive cows (71). Although infections in cattle appear mild, the outbreaks have led to additional infections in humans, other farm animals, and wildlife. In total, the U.S. has reported 70 human cases of H5N1. The first case was identified in 2022 associated with exposure to infected birds. Since April 2024 to May 2025, there have been 41 human cases in the U.S. related specifically to exposure to infected dairy cattle. Human infections have been clinically presented as a mild illness, with the most striking symptom being subconjunctival hemorrhage with concomitant thin, serous drainage (72–75). One human death in the U.S. has been linked to infection with H5N1; however, the person was over 65 years of age and had underlying health conditions (76). The other notable transmission has been from dairy cattle to farm cats. Cats likely infected by consuming raw milk from diseased cattle developed severe systemic illness, leading to mortality—a progression more severe than typically seen in humans or cattle (77). Clinical examination of infected cats indicated systemic disease with neurological and cardiac involvement characterized by ataxia, stiff body movements, oculonasal discharge, blindness, and viral antigen-positive tissues (brain, lung, heart, and retina) (77).

Investigations into the large outbreak in dairy cattle suggest that older, multiparous dairy cattle in the late lactation stage are most affected (70). The case reports describe nonspecific mild acute illness in infected cows clinically characterized by reduced feed intake, lethargy, respiratory secretions, slow rumination, temperature <41°C, anemia, leukocytopenia, and a significant decrease in milk production (28, 69, 77). The expressed milk from ill cows was thicker than typical milk and yellow, appearing like colostrum. Clinical signs peaked between 4 and 6 days after initial identification of illness, with resolution noted after 10 to 14 days. The drop in milk secretion lasted for 4 weeks even after recovering from clinical signs. Evaluation of the influenza virus-positive bovine mammary glands found neutrophilic multifocal lesions that were concomitantly positive for high viral NP within the mammary secretory alveolar epithelial cells. Less viral protein was found in the ductal epithelium (28, 56, 77). Viral RNA was detected at low Ct values (ranging from 12 and 23, depending on the target screened) in the mammary glands and expressed milk, indicating potentially high viral loads. In contrast other tissues, including those from the respiratory tract, showed inconsistent detection, with Ct values ranging from 30 and >40 in several naturally infected cows from outbreak farms (77).

### Experimental infection of HPAI H5N1 in lactating dairy cows, heifers, and calves

The routes of infection and transmission of the H5N1 virus among dairy cows and between herds remain unclear. To further explore the natural history of H5N1 infection in dairy cattle and potential transmission routes, experimental infection studies with clade 2.3.4.4b H5N1 have been performed. Baker and colleagues inoculated lactating Holstein cows and Holstein heifer calves with H5N1 clade 2.3.4.4b (A/dairy cattle/Texas/24008749-002/2024). The cows received an intramammary dose of 10^5^ TCID_50_ in two of the four quarters, while the calves were inoculated via aerosol inhalation at 2 × 10^6^ TCID_50_ (78). Following inoculation, lactating cows developed clinical signs consistent with those observed during herd outbreaks, including decreased rumination, lethargy, reduced food intake, dry or loose feces, nasal discharge, and discolored viscous milk. The most striking clinical feature was decreased milk production, which only recovered to ~70% of original milk yields by the end of the study (day 23 post-inoculation). Viral RNA (<25 Ct) was detected in expressed milk from the inoculated quarters as early as day 1 post-inoculation and persisted for 14 days. Infectious virus was concurrently detected in pooled milk samples through day 10 post-inoculation. Interestingly, low levels of viral RNA (>38 Ct) were inconsistently detected in milk from adjacent uninoculated quarters, while viral RNA was undetectable in blood and feces. Lactating cows were positive for NP antibodies on day 7 in serum and day 9 in milk. Virus-neutralizing antibodies were detected in both serum and milk from inoculated mammary glands as early as day 9 post-inoculation. Milk samples from uninoculated quarters were not positive for virus-neutralizing antibodies throughout the time course. Serum had a positive HAI of 1:80 and 1:160 for the two lactating cows on day 24 post-inoculation. Histopathological evaluation at the study endpoint revealed influenza A NP protein in the nuclei and cytoplasm of epithelial cells lining the secretory alveoli of the directly inoculated mammary glands (78). .

Aerosol inoculated heifers did not have overt signs of clinical disease presenting only with transient nasal secretions (78). Nasal, oropharyngeal, saliva, and ocular samples were collected and analyzed for viral RNA, but the results were inconsistent, showing low levels of viral RNA (>38 Ct). Additionally, the detection of infectious virus in these samples was also variable. Tissue samples were collected from two heifers on day 7 post-inoculation, while the other two heifers were monitored until day 20 post-aerosol inoculation. Both heifers sampled on day 7 had viral RNA-positive tissues, including the retropharyngeal lymph node, lung lobes, and nasal turbinates. Antibody analysis revealed low HAI and neutralizing antibody titers, which did not meet criteria for positivity. Minimal gross lung pathology was identified.

Halwe et al. also presented findings from experimental infections of lactating dairy cows with HPAI H5N1 virus. In this study, lactating dairy cows were intramammary-inoculated with either 10^6.1^ TCID_50_ of H5N1 B3.13 (A/Cattle/Texas/063224-24-1/2024) or 10^5.9^ TCID_50_ of EU genotype euDG H5N1 clade 2.3.4.4b (A/wild_goose/GermanyNW/00581/2024, H5N1 euDG). Intramammary infection with both viruses resulted in fever, lethargy, and reduced feed intake. In each group, two out of the three cows deteriorated into clinical conditions meeting humane endpoint criteria. After infection, milk yields dropped in all animals by over 90%. Milk tested positive for both viruses as early as 1-day post-infection, with viral genome loads peaking at 3 days post infection. Viral titers in the milk reached a peak of 10^8^ TCID_50_/mL, but no systemic infection was observed. Virus isolated from the mammary gland tissue showed persistence longer in some cows infected with B3.13 than with euDG, indicating possible slight differences in tissue tropism or viral clearance. Interestingly, the euDG strain showed a notable mutation (PB2 E627K) after intramammary infection, suggesting adaptation for mammalian replication. The PB2 E627K mutation is notable for its association with human infections caused by avian influenza viruses and has been specifically implicated in enhancing mammalian transmission of these viruses (79). A single amino acid in the PB2 gene of influenza A virus is a determinant of host range (80). This mutation was not observed in the B3.13 strain, which maintained its bovine-adapted PB2 M631L mutation. Pathological assessment of the lactating tissue showed acute epithelial necrosis and intraluminal cellular debris with intralesional antigen detection. No significant histological changes or viral antigen were observed in respiratory tissues. In cows, after intramammary inoculation with H5N1 strains, serum antibodies were detected by 7 days post-infection, and neutralizing antibodies appeared in milk starting from day 9. These antibodies reached a peak and remained elevated throughout the study period.

In addition, six calves were oronasally inoculated with the H5N1 B3.13 strain, which resulted in only mild respiratory symptoms such as nasal mucus secretion and occasional coughing, with no significant fever or systemic illness. Despite viral shedding from the nasal passages in five of the six calves for up to 8 days, no transmission occurred to sentinel calves housed with the infected animals. The virus was primarily detected in the upper respiratory tract, particularly in mucosa-associated lymphoid tissues, with no evidence of systemic spread or significant tissue damage. Although viral RNA was found in nasal and oral swabs, rectal and other bodily fluids remained largely negative. Importantly, the calves developed an immune response, producing influenza A-specific antibodies by 10–14 days post-infection.

Taken together, experimental inoculation studies in cattle suggest efficient infection and disease development when lactating cows are inoculated through the intramammary route, while respiratory tract exposure led to more variable results in respect to evidence for viral RNA, active virus replication, or development of clinical signs in the upper or lower respiratory tract.

### Studying bovine-origin H5N1 2.3.4.4b infection and transmission using small animal models

Understanding the mechanisms regulating H5N1 infection, transmission, and immune protection in dairy cattle will be essential to stopping the current outbreak and preventing future outbreaks. However, controlled cattle studies are challenging and labor-intensive due to the complexities of infecting large animals in a biosafety level 3 facility. Considering these issues, developing preclinical models for H5N1 2.3.4.4b mammary gland infection and transmission is essential to advance our understanding of such infections across species.

Mice and ferrets are standard preclinical models for studying influenza virus infection, transmission, and evaluation of therapeutics (26, 81–85). Guan and Eisfeld et al. utilized mice and ferrets to investigate the pathogenicity and transmission of the H5N1 2.3.4.4b dairy cattle virus in comparison to clade 1 H5N1 and seasonal H1N1 influenza virus infections (86). Oral inoculation of female BALB/c mice with milk from H5N1-infected dairy cattle led to an inconsistent number of mice experiencing significant weight loss and testing positive for virus in the respiratory tract and extrapulmonary organs (87). In contrast, intranasal inoculation with either the H5N1 2.3.4.4b dairy cattle virus or clade 1 H5N1 virus led to 100% mortality, with all mice showing infectious virus in the respiratory tract and some exhibiting infection in extrapulmonary tissues (86). This suggests that oral ingestion is not a robust mode of infection in mice. Female, non-lactating ferrets were also assessed for respiratory infection following intranasal inoculation with either clade 2.3.4.4b or clade 1 H5N1 influenza virus. Inoculations with both viruses led to similar trends in clinical data, as observed in infected mice with consistent viral replication in the respiratory tract and some infection in extrapulmonary organs including nonlactating mammary glands (86).

The investigators also assessed the potential for virus transmission from an intranasally infected lactating mouse to her co-housed pups. Pups housed with mothers infected intranasally with the H5N1 2.3.4.4b dairy cattle virus tested positive for infectious virus on days 7 and 9 post-maternal inoculation. In contrast, adult contact cage mates did not test positive, suggesting that virus transmission from the mother to pups likely occurred through nursing rather than via typical contact or aerosol routes. These findings were further supported by contact transmission studies in ferrets. Over a 13-day observation period, none of the recipient cage-mate ferrets tested positive for the virus after being housed with an H5N1 2.3.4.4b dairy cattle virus-infected donor ferret. Together, these findings suggest that the virus does not efficiently transmit through general contact between non-lactating ferrets but is transmitted from lactating female mice to pups, highlighting the mammary gland and nursing as critical factors in the current epizootic H5N1 spread among dairy cattle (86). In another study, C57Bl/6 mice were intraductally inoculated with A/bovine/Ohio.B24OSU-439/2024 (H5N1) influenza virus into actively lactating nipples (an average of seven lactating nipples per mouse). After intraductal infection, 50% of the mice exhibited weight loss ranging from 5% to 30%, with one mouse succumbing to infection. The deceased mouse had high viral titers in the mammary glands, lung, and brain, suggesting systemic viral spread (88). However, when murine mammary glands were inoculated with pandemic H1N1 virus A/California/04/2009, the virus failed to replicate in the mammary epithelium of mice, in contrast to the successful replication previously observed in the ferret (26). Furthermore, our group recently conducted a similar study using lactating ferrets to investigate the transmission of H5N1 clade 2.3.4.4b through milk, as well as its pathogenesis in both dams and their suckling kits (89). Mammary glands of lactating dams were inoculated with A/bovine/Ohio/B24OSU-342/2024 (H5N1) influenza virus at 2.5 weeks postpartum. Both kits and dams reached humane endpoints by 4 and 6 days post-inoculation, respectively. Viral RNA increased over time in milk, with mammary tissue exhibiting the highest viral RNA levels compared to other tissues. Viral RNA was also detected in kit stomach contents, lungs, bronchoalveolar lavage (BAL) fluid, as well as nasal and oral swabs. The viral kinetics observed in the oral and nasal cavities of kits suggest that infection likely originated from ingestion of H5N1-positive milk, followed by respiratory transmission back to the dam via close naso-oral contact. This study highlights the high susceptibility of lactating ferrets and their offspring to HPAI H5N1 infection and the mammary gland as a potential site of viral replication and milk-borne transmission.

Importantly, pre-existing influenza immunity influences outcomes of subsequent exposures, with preclinical models giving insights into the association of severe disease risk with sequential infections. Previously, we demonstrated that ferrets imprinted with a seasonal H1N1 virus were protected from challenge with a clade 1 H5N1 virus (90). Moreover, we recently showed that mice with pre-existing immunity to H1N1 were protected from H5N1 clade 2.3.4.4b-laden milk (91). Interestingly, repeated oral exposure using pasteurized milk containing inactivated H5N1 clade 2.3.4.4b virus did not prevent or accelerate mortality from lethal HPAI H5N1 challenge. However, it was associated with a ~ 0.5 log_10_ reduction in viral titers in the brain and delayed onset of clinical signs. Importantly, we have recently demonstrated that experimental animals (mice and ferrets) previously infected with seasonal H1N1 or vaccinated with a live-attenuated influenza vaccine (LAIV) were protected from lethal H5N1 clade 2.3.4.4b disease, potentially due to cross-reactive T cell responses, which supports findings from other studies (92, 93).

### Immunology of the mammary gland: implications for therapeutic strategies

The mammary gland represents a unique and understudied site for influenza virus replication. To reduce infection and disease in this tissue, it is critical to characterize the local immune response within the mammary gland following infection. Experimental intramammary infection of dairy cows with H5N1 influenza virus resulted in the detection of virus-neutralizing antibodies in milk as early as day 9 post-inoculation (28, 29). Moreover, cows intramammarily infected with H5N1 were protected from reinfection 31 days post-inoculation, coinciding with sustained virus-neutralizing antibody levels in milk (94). This mirrors findings from the 1950s, when cattle inoculated with H1N1 also exhibited protection upon re-exposure, as previously discussed. These findings suggest that intramammary infection with H5N1 virus in lactating dairy cows induces immunity either locally within the mammary gland or through serum-derived antibodies that transudate into the gland following systemic antigen stimulation. The durability of mammary gland immunity and the specific contributions of IgG and IgA to sustained protection are currently unknown.

Several reviews have summarized the immunology of the lactating mammary gland (95, 96). Antibody composition varies by species; in humans, IgA is the predominant isotype, comprising >75% of the total immunoglobulin pool (97, 98), and is primarily produced by local IgA-secreting plasma cells within the mammary tissue (99). Human breast milk also contains IgG, which is thought to be predominantly serum-derived. In contrast, IgG is the predominant isotype in dairy cow milk, largely derived from serum, with minimal contributions from IgA (97). In dairy cows, boosting systemic H5N1-neutralizing IgG through intramuscular vaccination is a rational strategy to increase H5N1-specific IgG levels in milk, given the naturally high concentration of IgG in bovine milk. In humans, however, a more nuanced approach may be necessary to protect both the lactating mammary gland and the breastfeeding infant during an emerging H5N1 outbreak. Mucosal vaccination strategies that stimulate both local IgA production within the mammary gland and systemic IgG responses, which can transudate into milk, are likely to be ideal. Further research is needed to identify vaccination strategies that promote anti-influenza immunity within the mammary gland and in milk.

Understanding the immune response to non-influenza viruses in the mammary gland may offer valuable insights into mammary gland susceptibility and broader antiviral defense mechanisms. Rotavirus and norovirus are leading causes of enteric infections, responsible for about 200,000 annual deaths, primarily in underdeveloped countries (100, 101). In a seminal study published in 2022, Ghosh and colleagues found that rotavirus and norovirus replicated in the salivary glands of suckling mouse pups. This resulted in the transfer of both viruses to the mammary gland, resulting in productive replication. Rotavirus and norovirus infection led to increased viral genomes in milk and the mammary gland, accompanied by a sharp rise in secretory IgA in both maternal milk and the pups' gut, peaking at day 5 and remaining elevated through day 14. These findings highlight two important implications: first, milk secretory IgA likely plays a crucial role in antiviral immune responses within both the mammary gland and neonatal intestines; second, a feedback mechanism between the mother and pups appears to trigger the increased secretory IgA levels in maternal milk. These results parallel the antibody responses seen in cows following HPAI H5N1 infection, where an increase in milk antibodies occurs in response to viral replication.

Although substantial research has been conducted on the transmission of CMV (39) and HIV-1 (102) from the mammary gland and breast milk to human infants, neither CMV nor HIV-1 appears to directly damage the secretory mammary epithelial cells, distinguishing these infections from viral mastitis-associated influenza virus infections observed in cows and small animal models. There is no direct evidence of influenza virus replication in the human mammary gland, and the U.S. Centers for Disease Control and Prevention recommends that breastfeeding should continue during maternal influenza infection (103). However, the potential for influenza viruses to infect human mammary glands remains poorly studied. Women’s health should continue to be a priority in this evolving field.

## A ONE HEALTH VIEW: THE FUTURE OF CLADE 2.3.4.4b H5N1 IN DAIRY CATTLE, AVIAN SPECIES, AND OTHER MAMMALS

The current clade 2.3.4.4b H5N1 outbreak in dairy cattle represents a complex One Health issue, impacting public health, the livestock industry, and wildlife. Specifically, infection in cows reduces production yields and profits for producers, while also increasing economic and healthcare costs associated with managing sick cattle. Additionally, the large volume of contaminated milk increases the risk of spread to wildlife, farm animals, dairy workers, and individuals involved in milk processing (77). Ongoing infections and transmission events in dairy cattle and other species heighten the risk of viral mutations that enhance its ability to infect and spread among humans or livestock, raising concerns for pandemic preparedness and the potential establishment of an enzootic reservoir in livestock populations.

Although pasteurization is demonstrated to inactivate H5N1 influenza virus (104, 105), the detection of viral genomic fragments in retail dairy products has raised concerns within the dairy industry that consumer fears could lead to decreased demand (106, 107) (communication with the Dairy Farmers of Ontario, Canada, and Ohio, USA). It remains unclear why viruses in this clade have been so successful at persisting and spreading among wild migratory birds while also gaining the ability to infect previously unassociated mammalian hosts, such as polar bears (108). Alarmingly, H5N1 2.3.4.4b has caused significant mortality in pinnipeds, including over 17,000 southern elephant seals in Argentina (109) and 811 sea lions in a single colony over 2 months (110).

**Fig 2:**
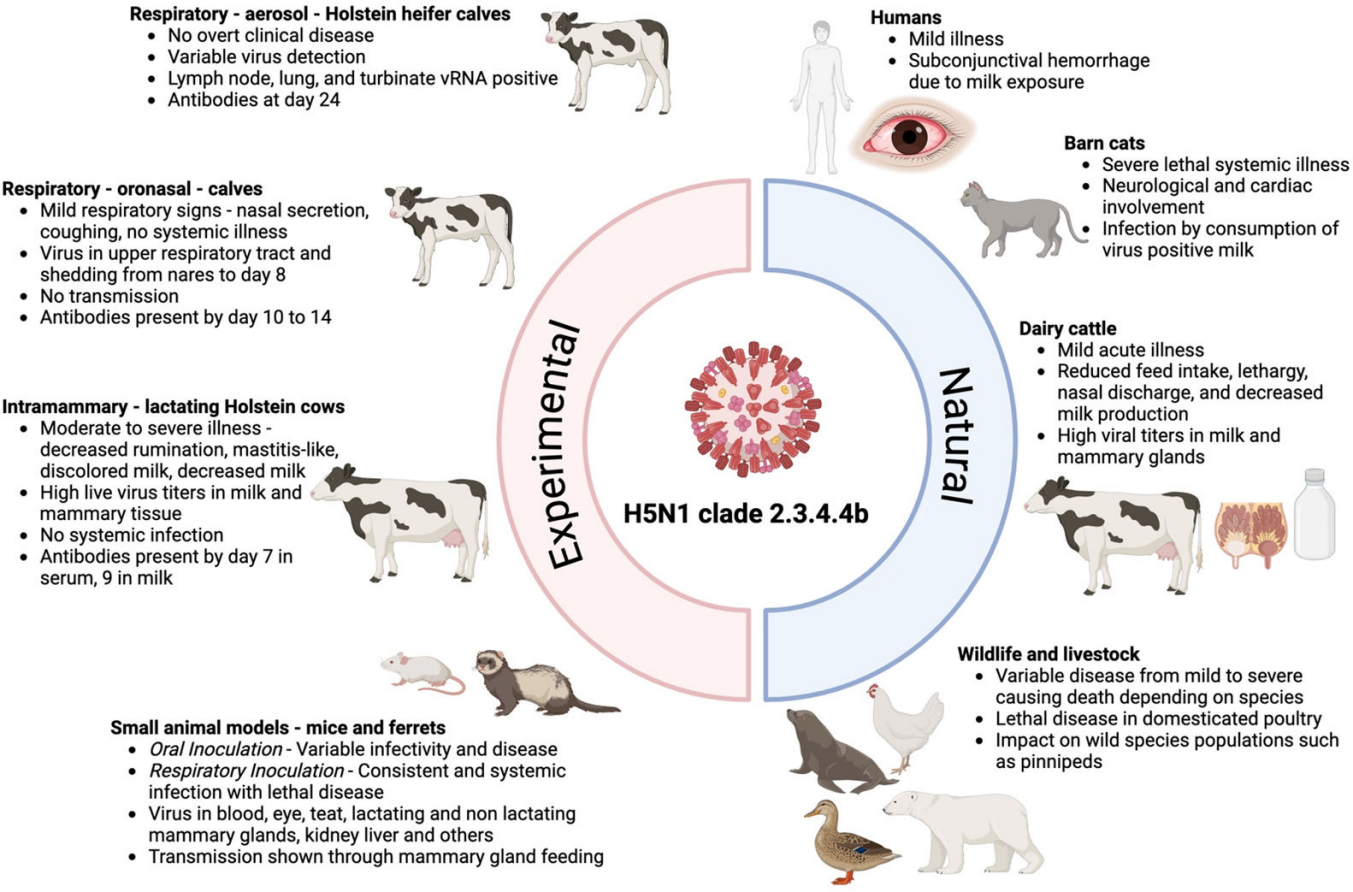
Summary of experimental and natural H5N1 clade 2.3.4.4b infection in wildlife, livestock, and small animal models.

In the context of the recent H5N1 clade 2.3.4.4b outbreak in dairy cattle, the U.S. Department of Agriculture has determined that milk from infected cows is the primary source of virus transmission to other dairy cattle, animals, and humans (111). Preliminary studies suggest that contaminated milking equipment, as well as infected air and water, may play a role in transmission between cows (112, 113). Additionally, the geographic spread of the virus may be facilitated by the movement of infected dairy cattle between farms, including across state lines (114). Data from experimental and case reports (Fig. 2) indicate that lactating dairy cows shed the highest viral titers in milk, while shedding from the respiratory tract remains low, suggesting that respiratory transmission may not play a significant role in virus spread; however, further research is needed. If mammary gland infection and transmission are the primary drivers of the dairy cattle outbreak, prevention methods targeting the milking process—such as enhanced disinfection of milking equipment or improved worker personal protective equipment—may be more effective and feasible than strategies aimed at controlling airborne transmission. Further research is essential to clarify the site and mechanisms of viral replication within the mammary gland and to understand the potential implications for lactating humans.
